# The role of miRNAs in the diagnosis of stable atherosclerosis of different arterial territories: A critical review

**DOI:** 10.3389/fcvm.2022.1040971

**Published:** 2022-11-25

**Authors:** Ana Rita Teixeira, Vera Vaz Ferreira, Tiago Pereira-da-Silva, Rui Cruz Ferreira

**Affiliations:** ^1^Department of Cardiology, Hospital de Santa Marta, Centro Hospitalar Universitário de Lisboa Central, Lisbon, Portugal; ^2^NOVA Medical School | Faculdade de Ciências Médicas, Universidade NOVA de Lisboa, Lisbon, Portugal

**Keywords:** carotid, coronary, diagnostic biomarker, lower limbs, miRNAs, multi-territorial, stable atherosclerosis

## Abstract

Atherosclerotic disease is a major cause of morbidity and mortality worldwide. Atherosclerosis may be present in different arterial territories and as a single- or multi-territorial disease. The different phenotypes of atherosclerosis are attributable only in part to acquired cardiovascular risk factors and genetic Mendelian inheritance. miRNAs, which regulate the gene expression at the post-transcriptional level, may also contribute to such heterogeneity. Numerous miRNAs participate in the pathophysiology of atherosclerosis by modulating endothelial function, smooth vascular cell function, vascular inflammation, and cholesterol homeostasis in the vessel, among other biological processes. Moreover, miRNAs are present in peripheral blood with high stability and have the potential to be used as non-invasive biomarkers for the diagnosis of atherosclerosis. However, the circulating miRNA profile may vary according to the involved arterial territory, considering that atherosclerosis expression, including the associated molecular phenotype, varies according to the affected arterial territory. In this review, we discuss the specific circulating miRNA profiles associated with atherosclerosis of different arterial territories, the common circulating miRNA profile of stable atherosclerosis irrespective of the involved arterial territory, and the circulating miRNA signature of multi-territorial atherosclerosis. miRNAs may consist of a simple non-invasive method for discriminating atherosclerosis of different arterial sites. The limitations of miRNA profiling for such clinical application are also discussed.

## Introduction

Atherosclerotic disease is highly prevalent and, despite the recent advances in the therapeutic field, it is still accountable for considerable cardiovascular morbidity and mortality worldwide (1). According to atherosclerosis location, it may present as a localized, single-territorial disease of different arterial territories or a systemic, multi-territorial disease (2). Little is known on the mechanisms that regulate the development of atherosclerosis in different arterial territories and the atherosclerosis extent to single or multiple arterial beds (3, 4). The different phenotypes of atherosclerosis are not only attributable to acquired cardiovascular risk factors and genetic Mendelian inheritance (5, 6). Post-transcriptional regulators, including miRNAs, also contribute to such heterogeneity (4, 7).

miRNAs, non-coding molecules of ribonucleic acid (RNA) of about 20 nucleotides, have received most of the attention during the last decades for their role as dynamic regulators of gene expression in different biological processes (8). They regulate the gene expression at the post-transcriptional level by binding to the target messenger RNA (mRNA), preventing its expression or inducing its degradation (9, 10). Most miRNAs are transcribed from DNA sequences into primary miRNAs (pri-miRNAs) and processed into precursor miRNAs (pre-miRNAs) and mature miRNAs (9). Generally, miRNAs interact with the 3′ UTR of target mRNAs to suppress expression, even though interaction with other regions, including the 5′ UTR, coding sequence, and gene promoters, have also been described (10). miRNAs regulate many biological processes associated with atherogenesis, including cell growth, proliferation, differentiation, migration, senescence, apoptosis, and angiogenesis (11–14). Besides their biological role, miRNAs can be used as diagnostic biomarkers and several studies reported dysregulated levels of circulating miRNAs in patients with atherosclerosis.

Atherosclerosis expression, including the associated molecular phenotype, varies according to the specific arterial territory. In fact, the composition of the atherosclerotic plaque and the levels of different circulating biomarkers vary according to the involved arterial territory (15, 16). Therefore, the circulating miRNA expression profile may vary also according to the involved arterial territory. In this review, after discussing the role of miRNAs in atherosclerosis regulation, we present a critical overview of the specific circulating miRNA profiles associated with atherosclerosis of different arterial territories, the common circulating miRNA profile of stable atherosclerosis irrespective of the involved arterial territory, and the circulating miRNA signature of multi-territorial atherosclerosis.

This review follows the work previously carried out by our research group (15), which assessed the circulating miRNA profiles in atherosclerosis of the carotid, lower extremity, and renal arteries, but not the coronary arteries. This is a more comprehensive review, considering that: atherosclerosis of the coronary territory is covered in this review; recently published articles of circulating miRNAs in coronary and non-coronary territories, which have not been included in prior reviews, are addressed in this review; and the miRNAs expression profile of multi-territory atherosclerosis is included. Of note, a critical analysis of the miRNAs' potential as diagnostic biomarkers of atherosclerosis of different territories, based on pre-established criteria, is presented.

These circulating miRNA profiles may provide clues to the underlying pathophysiology of atherosclerosis of different arterial territories. Importantly, such dysregulated miRNAs are potential non-invasive diagnostic biomarkers of stable atherosclerosis of different vascular beds. To our knowledge, this is the first overview of circulating miRNA profiles according to atherosclerosis of different territories, including the coronary, carotid, lower limbs, renal, and multi-territorial atherosclerosis. A critical analysis regarding robustness of results, based on predefined quality criteria, is presented.

## The role of miRNAs in the regulation of atherosclerosis

There is growing evidence that different miRNAs are associated with the mediation of key cellular and molecular processes involved in the pathophysiology of atherosclerosis, including atherogenesis and atherosclerotic plaque progression and rupture (11–14, 17–20). An important concept is that some miRNAs participate in multiple pathways and in different steps of a specific pathway, acting as post-transcriptional hubs (11–14, 17, 18). Furthermore, each miRNA may exert atheroprotective effects, proatherogenic effects, or both, which highlights the complexity of atherosclerosis regulation by miRNAs (11–14, 17, 18). The role of miRNAs in the regulation of endothelial cell, monocyte–macrophage, and vascular smooth muscle functions, cholesterol homeostasis, and extracellular matrix composition are discussed below.

**Figure 1:**
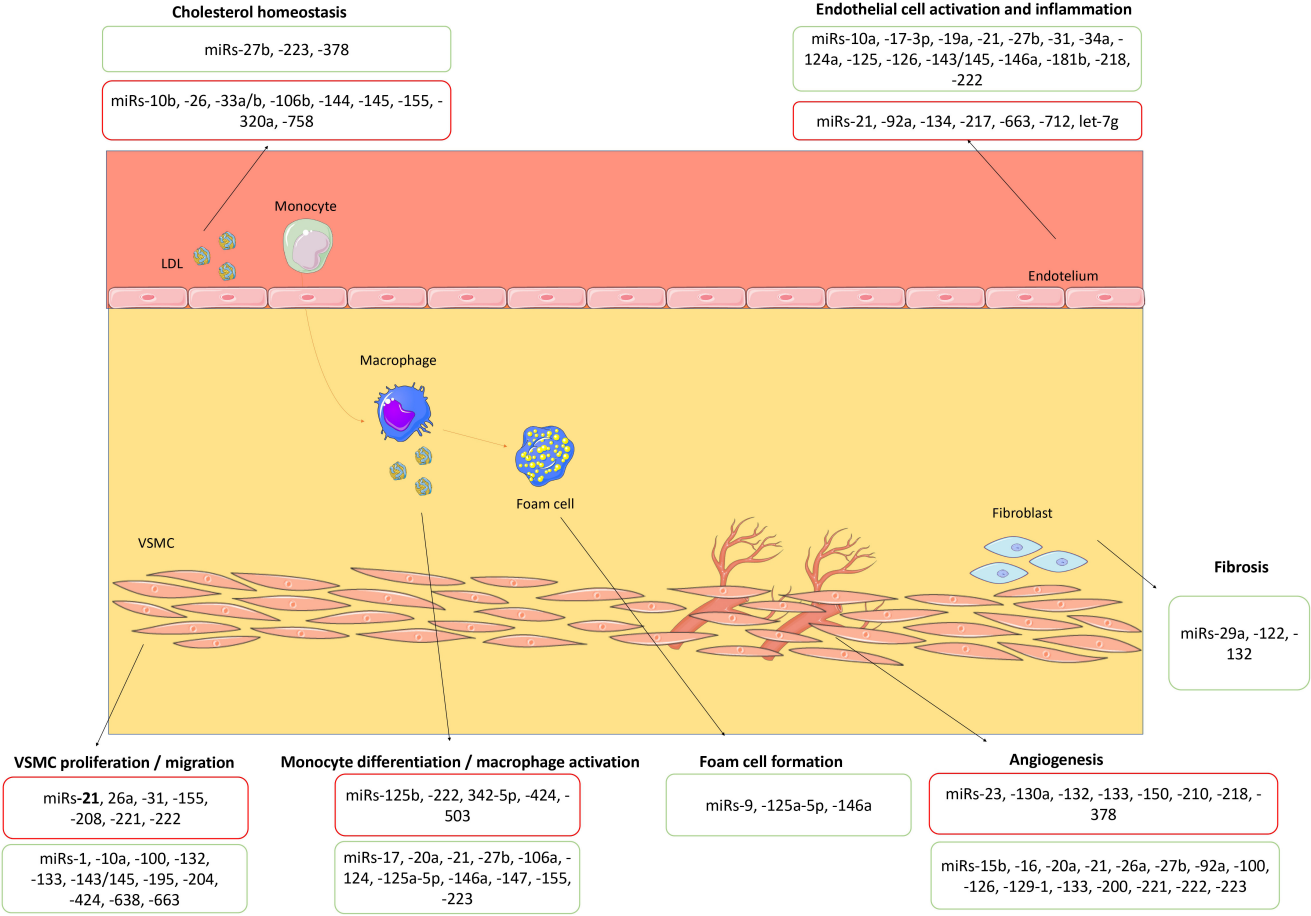
Biological roles of miRNAs in atherosclerosis's pathophysiology. miRNAs with proatherogenic and atheroprotective effects are highlighted in red and green boxes, respectively. VSMC, vascular smooth muscle cell. The figure was partly generated using Servier Medical Art, provided by Servier, licensed under a Creative Commons Attribution 3.0 unported license (https://creativecommons.org/licenses/by/3.0/).

The first steps in atherosclerosis development include endothelial dysfunction, followed by inflammatory response and foam cell formation (14). Endothelial cells play an essential role in the occurrence of vascular diseases, particularly atherosclerosis, as they regulate the synthesis and secretion of vasoactive substances, platelet aggregation, leukocyte adhesion, and thrombosis, among other functions (14). In fact, endothelial dysfunction is considered an early marker of atherosclerosis development (14). Different miRNAs, such as miR-21 (21), miR-126 (22–24), miR-146a (25) and miR-221 (26), regulate endothelial cell senescence, proliferation, and migration and influence inflammatory pathways initiated in endothelial cells. Members of the miR-181 family have been shown to suppress the inflammatory response of endothelial cells by targeting mediators of NF-kB pathways (27–29). Also, miR-31 and miR-17-3p are involved in a negative feedback loop that directly inhibits the expression of the adhesion molecules (30). Some miRNAs, including miR-34a (31), miR-217 (32) and miR-146a (33) have important roles in the endothelial aging, which is intimately involved in alterations in the biomechanical and structural properties of the vascular endothelial cells. Interestingly, several miRNAs, including miR-10a (34), miR-19a (35), miR-23b (36), miR-101 (37), and miR-143/145 (38), are inducible by high shear stress and exert atheroprotective effects. Some miRNAs, including miR-21 (13, 21), miR-27b (39), and miR-218 (40), specifically modulate angiogenesis by targeting endothelial cells. Other miRNAs, such as miR-126 (41), miR-221 and miR-222 (42, 43), can also regulate angiogenesis development. Of note, the overexpression of miR-92a (44), miR-129-1 and miR-133 can block this process (45). Angiogenesis facilitates the growth of atherosclerotic lesions and, within the plaques themselves, plays a crucial role in plaque destabilization and rupture. Monocyte–macrophage activity is also regulated by miRNAs (14). In the early stages of atherogenesis, monocytes infiltrate into the sub-endothelium and differentiate into macrophages, uptake low-density lipoprotein (LDL) cholesterol and transform into foam cells, which promote the inflammatory immune responses and plaque formation (4). They also release chemokines and cytokines, that induce acute inflammatory reaction and hereby promote atherosclerosis progression (4, 14). Among other miRNAs, miR-21 (46) and miR-146a (47) modulate monocyte–macrophage activity, including monocyte differentiation, macrophage phenotype, and macrophage-mediated inflammation. MiR-21 is the most abundant miRNA in macrophages, and its overexpression has an anti-inflammatory role (18). MiR-146a does not only decrease the lipid uptake in macrophages, but also inhibits endothelial activation by promoting endothelial nitric oxide synthase expression (8). MiR-125a-5p can also play a protective role in atherosclerosis by regulating the pro-inflammatory response and the uptake of lipids by macrophages (48). On the other hand, miR-155 is crucial for the classical activation of macrophages and expression of inflammatory mediators (49). Vascular smooth muscle cell proliferation, migration, and phenotype influence the composition of the atherosclerotic plaque and are also highly regulated by miRNAs, including miR-21 (13, 14). In experimental models of neointima, there is overexpression of miR-21, miR-146, miR-214, miR-221 and miR-352, whereas miR-125a, miR-125b, miR-133, miR-143, miR-145, and miR-365 are downregulated (13, 17). Systemic cholesterol homeostasis and the LDL inflow into the arterial wall affect the atherosclerotic plaque development and are regulated by different miRNAs, particularly miR-27b (11). Beyond this, miR-33a and miR-33b were also described as key regulators of cholesterol and fatty acid homeostasis, being overexpressed in atherosclerosis (50). Finally, the extracellular matrix composition of the atherosclerotic plaque, including the fibrotic tissue, is associated with the atherosclerotic plaque size and its stability (20); miR-29 is one of the miRNAs that modulate the fibrotic content of the atherosclerotic plaque (51). Therefore, the pathological basis of atherosclerotic plaque formation and development is highly regulated by miRNAs (Figure 1). There are gaps in evidence regarding the regulation of atherosclerosis by miRNAs. In fact, some miRNAs were reported to be dysregulated in atherosclerotic disease, such as miR-363, although their role is not well established *in vitro* yet. Furthermore, some miRNAs, such as miR-132, may present both proatherogenic and atheroprotective effects and the underlying mechanisms that explain such heterogeneity of effects of each miRNA are not entirely known.

## miRNAs as potential biomarkers of stable atherosclerosis

Intracellular miRNAs are released into body fluids, including peripheral blood, and circulating miRNAs remain stable in serum and other body fluids because they are protected from degradation by endogenous RNases (52). Some researchers hypothesized that the stability of circulating miRNAs may be achieved by the high stability of the Ago2 protein and Ago2-miRNA complexes (binding of miRNA to carrier molecules) (53) or due to the loading into lipoprotein complexes (such as HDL) or extracellular vesicles (such as exosomes and microvesicles), and the quantity of miRNA is crucial for its related functional impact (54). Importantly, the high stability of circulating miRNAs may facilitate their use as biomarkers of different diseases, including atherosclerosis. Of note, caution should be taken when assessing the EV-associated nucleic acids function since isolation methods influence the yield of recovery of EV-derived RNA (54).

As aforementioned, the molecular phenotypes of atherosclerosis vary according to the specific arterial territory involved, including the circulating miRNA profiles. Major territories of atherosclerosis include the coronary, carotid, lower limbs, and renal arteries. Moreover, atherosclerosis may be expressed as a single- or multi-territorial disease. The circulating miRNAs profiles for each scenario are herein discussed and a critical overview of robustness of results is presented, based on the reproducibility between studies, validation methods, adjustment in multivariate analysis, and diagnostic accuracy. The selection of studies was based on the knowledge of experts in the field. Of note, data of major systematic reviews were analyzed, including those focused on miRNA profiles in coronary and non-coronary atherosclerosis. Importantly, we focused on stable atherosclerosis but not acute ischemic events considering that the pathophysiology of stable atherosclerosis is associated with the initiation and expression of stable lesions rather than plaque instability and activation of the thrombotic cascade (2–5). We selected studies that assessed miRNAs isolated from serum and plasma, but not from blood cells, since the former are more likely to be used as biomarkers in the near future. The change in expression of miRNAs refers to altered circulating levels of miRNAs as assessed by RT-PCR assays. To evaluate the diagnostic accuracy of miRNAs and their ability to discriminate the presence of atherosclerosis we assessed the area under the ROC curve.

### miRNAs in coronary artery disease

For coronary artery disease (CAD) two systematic reviews reported a miRNA signature, for diagnostic purposes (55, 56). However, the results were based on both acute and stable atherosclerosis, which limits the interpretation of results considering the heterogeneity of included samples. Cardiac-enriched miRNAs, which are expressed abundantly in the heart and play crucial roles in cardiac physiology, were consistently dysregulated in CAD (miR-1, miR-133a, miR-208a/b, and miR-499) compared to controls (55, 56). In addition, the let-7 family, miR-92a, miR-145, miR-142, miR-17 and miR-126, which have a known role in cardiovascular pathophysiology, were also dysregulated in patients with CAD, with high reproducibility of the results (over 10 studies for each miRNA) (25). The identified miRNAs were found to regulate endothelial function and angiogenesis (miR-1 and miR-133), vascular smooth muscle cell differentiation (miR-133 and miR-145), communication between vascular smooth muscle cells and endothelial cells to stabilize plaques (miR-145), apoptosis (miR-1, miR-133, and miR-499), cardiac myocyte differentiation (miR-1, miR-133, miR-145, miR-208, and miR-499), and cardiac hypertrophy (miR-133) (18).

In studies focused on stable CAD, but not acute coronary syndrome, miR-145 (57–59), miR-29b (60), let-7 family (57–59), miR-92a (57, 61) and miR-126 (57, 58, 61, 62) were the most frequently downregulated miRNAs (57, 61, 63, 64), while miR-208, miR-215, miR-487a and miR-502 were up-regulated (60). There was higher reproducibility of results, with replication in different studies for miR-126 (57, 61, 63, 64), miR-145 (57–59), miR-92a (57, 61) and let-7 (58, 63). miR-155 (57, 58, 62) and miR-17 (57, 60, 65) were up-regulated in some studies and down-regulated in others. Importantly, levels of miR-215, miR-502, miR-487, and miR-29b were exclusively dysregulated in stable CAD (and not dysregulated in acute coronary syndrome), and therefore could be regarded as more specific biomarkers of stable CAD. Of note, the levels of some miRNAs are associated with the severity of CAD. In fact, the Gensini score was positively correlated with miR-155 (62) and miR-17-5p (65) expression levels and negatively correlated with miR-145 (58) and miR-126, miR-210 and miR-378 expression levels (61).

Regarding the aforementioned studies that report miRNAs' dysregulation in stable CAD, most were case-control studies (57–61, 63–65). The majority of studies defined CAD as obstructive atherosclerosis (stenosis of at least 50% of one major epicardial artery) on invasive coronary angiography, while controls presented no atherosclerosis (58–60, 62–64). One study defined obstructive CAD as an angiographical stenosis of at least 75% and compared with controls presenting non-significant CAD (65). In four studies, the results were obtained in a derivation cohort and prospectively tested in a validation cohort (57, 59–61). Those studies reported the dysregulation of miR-126 and miR-92a (57, 61), miR-17, miR-155, miR-145 (57), miR196a-5p, miR-3613-3p, miR-145-3p, miR-190a-5p (59), miR-17-5p, miR-210, miR-378 (61) miR-487a, miR-29b, miR-502, miR-208 and miR-215 (60). Of note, most studies of stable CAD (57, 61–65) had a targeted selection of miRNAs while the others had unbiased selection (58–60). Therefore, there may be a bias toward reporting popular miRNAs, considering the candidate-driven approaches.

Regarding adjustments for covariates, after adjustment for LDL-cholesterol, HDL-cholesterol, smoking, statins, angiotensin converter enzyme inhibitors, beta blocker, and calcium channel blockers, logistic regression indicated that lower levels of miR-155, miR-145 and let-7 were independently associated with stable CAD (58).

Some studies reported the area under the curve (AUC) of specific miRNAs to detect stable CAD. The AUC was 0.654 (95% confidence interval [CI]: 0.536–0.773) for let-7, 0.620 (95% CI: 0.491–0.747) for miR-155 and 0.670 (95% CI: 0.556–0.785) for miR-145 to detect CAD, whereas miR-92a showed a very low diagnostic accuracy, with an AUC of 0.520 (95% CI: 0.393–0.646). Interestingly, the combination of let-7, miR-155 and miR-145 resulted in a higher AUC value (0.708, 95% CI: 0.600–0.811), increasing the diagnostic accuracy (58). The AUC of miR-126, miR-17-5p, miR-92a, miR210 and miR-378 for predicting CAD was 0.641 (95% CI: 0.564–0.719), 0.609 (95% CI: 0.526–0.692), 0.622 (95% CI: 0.541–0.702), 0.617 (95% CI: 0.537–0.698) and 0.574 (95% CI: 0.492–0.656), respectively. When combining these five miRNAs, the AUC was 0.756 (95% CI: 0.687–0.725), which indicated a reasonable diagnostic accuracy (61). Of note, miR-17-5p presented an AUC of 0.894 (95% CI: 0.780–0.968) in one study (65). The AUC for combination of miR-487a, miR-502, miR-208 and miR-215, miR-29b was 0.850 (95% CI: 0.734–0.966, *p* < 0.001) and 0.909 (95% CI: 0.858–0.960; *p* < 0.001) in training and validation set, respectively (65). Individually, miR-29b (AUC of 0.867; 95% CI: 0.795–0.939) and miR-208 (AUC of 0.886; 95% CI: 0.811–0.941) were better than other miRNAs in discriminating atypical coronary artery disease from controls (60). MiR-196a-5p, miR-3613-3p, miR-145-3p, and miR-190a-5p had had good sensitivity and specificity for distinguishing patients with very early-onset CAD, with an AUC of 0.824 (95% CI: 0.731–0.917), 0.758 (95% CI: 0.651–0.864), 0.753 (95% CI: 0.643–0.863) and 0.782 (95% CI: 0.680–0.884), respectively (59).

### miRNAs in carotid artery disease

In carotid atherosclerosis, miR-21 (66, 67), miR-21-5p (68), miR-29c (69), miR-29a (70), miR-92a (71), miR-211 (67), miR-218 (67) and let-7 (72) were upregulated and miR-30 (73), miR-31 (67), miR-125a-5p (68), miR-126-3p (68), miR-143 (74), miR-145 (74), miR-221 (66), miR-221-3p (68), miR-222-3p (68) and miR-320b (75) downregulated. More recent studies added some data regarding miRNA profiles in carotid atherosclerosis, including the upregulation of miR-342-5p (76), miR-27a (77), and miR-146a (78) and downregulation of miR-181b (77) and miR-503-5p (79).

In most of the studies, the presence of atherosclerosis was assessed by carotid intima-media thickness (CIMT) measured by ultrasonography. Subclinical atherosclerosis was defined as CIMT 0.9–1.2 mm, whereas atherosclerosis was defined as CIMT ≥1.2 mm (68–73). In other studies, carotid atherosclerosis was defined as a carotid stenosis of at least 50% or carotid occlusion, either by ultrasound or angiography (66, 76).

The use of a derivation cohort followed by a validation cohort was reported in a study that assessed the expression levels of miR-21, miR-211, miR-218 and miR-31 (67).

Regarding adjustments for covariates, CIMT was associated with miR-29c levels (β = 0.529, 95% CI: 0.354–0.812; *p* < 0.001) adjusting for age, body mass index, systolic blood pressure, total cholesterol, and fasting blood-glucose (69). Adjusting for the traditional risk factors (including age, sex, hypertension, diabetes, hyperlipidemia, and smoking history), miR-21 upregulation and miR-221 downregulation were significantly associated with carotid artery disease severity, which was divided into three categories: absence of atherosclerosis, presence of atherosclerosis and clinical history of stroke (miR-21: adjusted *p* = 0.0047 and miR-221: adjusted *p* < 0.0001) (66). There was also an independent association between elevated miR-30 expression levels and CIMT in multivariate logistic regression analysis, adjusting for age, sex, body mass index, blood lipid, and blood pressure (β = −0.748, 95% CI: 0.278–0.806; *p* = 0.006) (73). Multiple linear regression analysis demonstrated that the expression of let-7 was independently associated with CIMT (β = 0.031, 95% CI: 0.015–0.047, *p* < 0.001) (72).

Regarding the diagnostic accuracy of miRNAs, miR-342-5p and miR-126-3p presented good discriminatory accuracy for detecting carotid atherosclerosis, with an AUC of 0.905 (95% CI not reported) and 0.888 (95% CI: 0.819–0.958), respectively (68, 76). MiR-181b and miR-27a presented reasonable accuracy, with an AUC of 0.791 (95% CI: 0.692–0.904) and 0.750 (95% CI: 0.651–0.885), respectively (77). Combined with traditional risk factors, miR-320b showed an AUC of 0.834 (95% CI: 0.765–0.903) (75), miR-21 an AUC of 0.90 (95% CI: 0.85–0.93) and miR-221 showed an AUC of 0.91 (95% CI: 0.87–0.94) (66). The combination of miR-21 and miR-21 presented a reasonable accuracy, with an AUC of 0.77 (95% CI: 0.72–0.82) (66). Combined with C-reactive protein, miR-29c presented an AUC of 0.900 (95% CI: 0.857–0.944) (69), in the diagnosis of carotid atherosclerosis. Individually, miR-29c also showed a good accuracy with an AUC of 0.870 (95% CI: 0.818–0.922) (69). The receiver operating characteristic curve showed that miR-146a may have some value for predicting plaque vulnerability in patients with carotid atherosclerosis (AUC of 0.64; 95% CI: 0.613–0.769) (78).

### miRNAs in lower limb artery disease

Regarding lower limbs atherosclerosis, miR-21 (80), miR-130a (81), miR-210 (80, 81), miR-124, miR-221-5p, miR-4284 (82) miR-411 (81), miR-4739 (83), miR-1827 (84), and miR-124-3p (85) were upregulated, while let-7 (86, 87), miR-15a (87), miR-15b, miR-16, miR-20b, miR-25, miR-26b, miR-28-5p, miR-126, miR-195, miR-335, miR-363 (86), miR-27b (81), miR-196b (87), miR-18a-5p, miR-30e-5p, miR-106a-5p, miR-223-3p, miR-652-3p (88), miR-221-3p, miR-432, miR-4306, and miR-4463 (82) were downregulated. One study showed a downregulation of miR-27b (76), while in other studies it was upregulated (80, 81).

Lower limbs atherosclerosis was defined as the presence of two major symptoms, intermittent claudication, and ischemic rest pain as the main criteria, complemented in some studies with arterial stenosis/occlusion identified on ultrasound imaging or angiography.

A derivation cohort followed by a validation cohort were used in studies that reported the dysregulation of let7e, miR-15b, miR-16, miR-20b, miR-25, miR-26b, miR-27b, miR-28-5p, miR-126, miR-195, miR-335, miR-363 (86) miR-124, miR-221-5p, miR-4284, miR-221-3p, miR-432, miR-4463, miR-4306 (82) and miR-4739 (83). MiR-1827 was evaluated in 3 phases: discovery, confirmation, and validation (84).

Levels of miR-4739 were assessed in patients with type 2 diabetes mellitus, adjusting for diabetes duration, hypertension, smoking, HbA1c, creatinine and total cholesterol (83). In the multivariate analysis, miR-4739 levels were independently associated with critical limb ischemia in patients with diabetes (OR = 12.818, 95% CI 1.148–143.143, *p* = 0.038) (83).

Regarding the diagnostic accuracy, miR-16 (AUC 0.93; 95% CI: 0.86–1.00; *p* < 0.001), miR-363 (AUC 0.93; 95% CI: 0.85–1.00; *p* < 0.001), and miR-15b (AUC 0.92; 95% CI: 0.82–1.00; *p* < 0.001) presented the highest AUC values. miR-195 (AUC 0.89; 95% CI: 0.79–1.00, *p* < 0.001), miR-126 (AUC 0.88; 95% CI: 0.77–0.98, *p* < 0.001), miR-27b (AUC 0.87; 95% CI: 0.75–0.99; *p* < 0.001), miR-28-5p (AUC 0.86; 95% CI: 0.75–0.97, *p* < 0.001), miR-26b (AUC 0.77; 95% CI: 0.62–0.93; *p* < 0.001), miR-335 (AUC of 0.76; 95% CI: 0.61–0.91, *p* < 0.001), miR-25 (AUC 0.75; 95% CI: 0.59–0.91; *p* = 0.01), let-7e (AUC 0.71; 95% CI: 0.55–0.88; *p* = 0.02), and miR-20b (AUC 0.69; 95% CI: 0.52–0.86; *p* = 0.04) presented good or reasonable diagnostic accuracy (86).

### miRNAs in renal artery disease

Regarding renal artery atherosclerosis, one study reported an upregulation of miR-126 (89). Data for this territory are less robust considering that it refers to a single study with a relatively small sample.

### miRNAs in atherosclerosis of different territories

**Figure 2:**
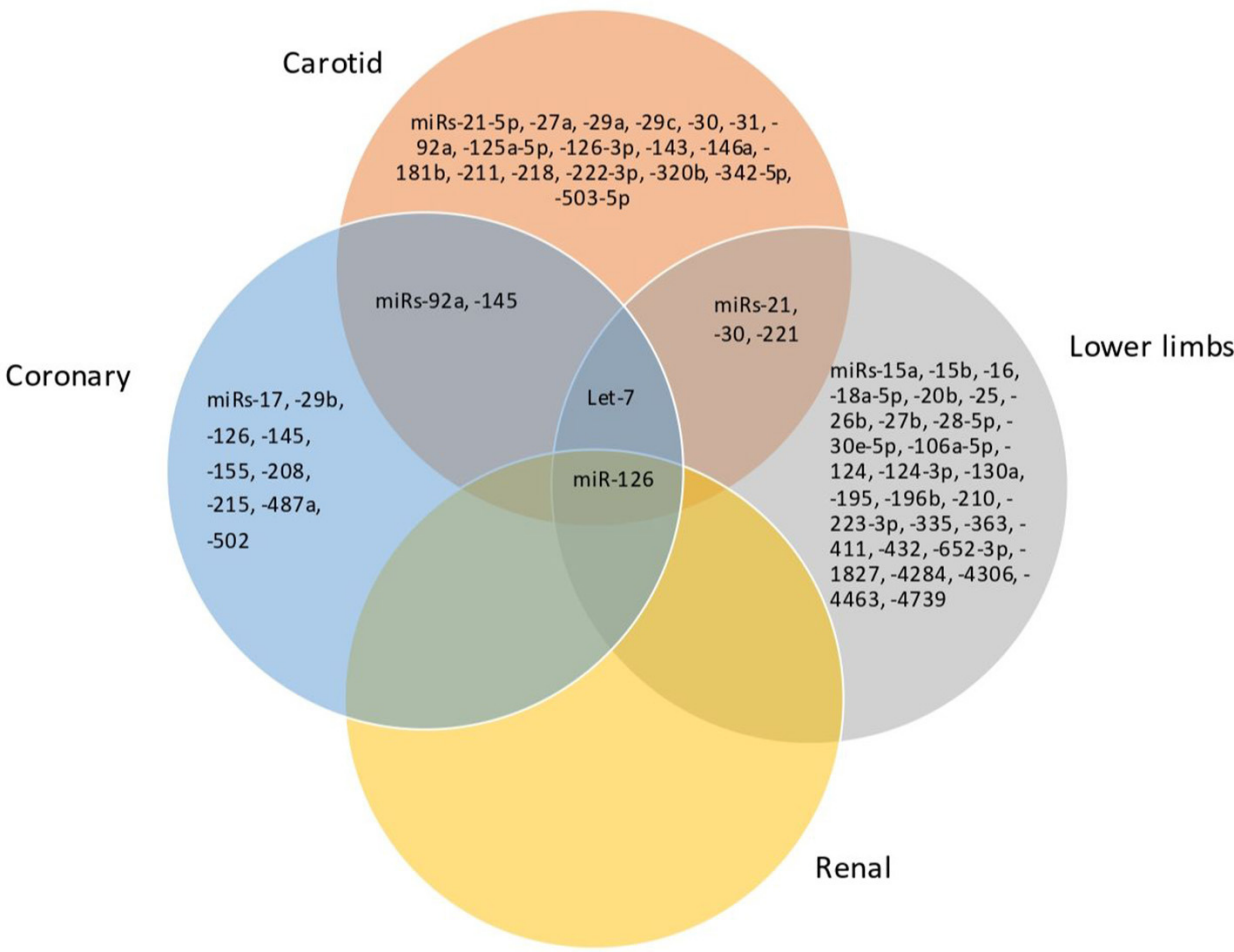
Venn diagram showing the miRNAs shared between carotid, coronary, lower limbs, and renal territories.

Considering the miRNAs with the most robust dysregulation in each territory, as discussed before, there are similar trends of dysregulation for the same miRNA in atherosclerosis of different arterial territories, particularly the coronary, carotid and lower limbs atherosclerosis (Figure 2). For instance, miR-126 was downregulated in coronary, carotid and lower limbs atherosclerosis, consisting thus in a common pattern of miRNA dysregulation in atherosclerosis of different territories. miR-126 was reported to be upregulated in renal atherosclerosis, although data are less robust for this territory as aforementioned. In addition, there was a common profile for atherosclerosis of the carotid and lower limbs arteries, consisting of an upregulation of miR-21 and downregulation of miR-30 and miR-221-3p. miR-145 was downregulated in both coronary and carotid territories.

### miRNAs in single- and multi-territorial atherosclerosis

Multi-territorial is frequently encountered in clinical practice and is associated with higher morbidity and risk of mortality compared with single-territorial atherosclerosis (16, 90). Multi-territorial atherosclerosis may have a different pathophysiology than that of single-territorial atherosclerosis and circulating miRNA profiles may also differ (16, 89). One study described the circulating miRNA expression profile in single- and multi-territorial atherosclerosis (90). Specifically, lower expression levels of miR-27b and miR-146 were independently associated with the presence of multi-territorial atherosclerosis involving the coronary, lower limbs, and carotid territories, after adjusting for other clinical and laboratory parameters (91). Moreover, a downregulation of miR-27b or miR-146 was associated with higher severity of atherosclerosis in different arterial territories (91). Both miRNAs showed reasonable accuracy for predicting severe systemic atherosclerosis with involvement of the three territories (AUC 0.76, 95% CI 0.60–0.91, *p* = 0.004 for miR-27b; and AUC 0.75, 95% CI 0.59–0.91, *p* = 0.009 for miR-126) (91). The expression levels of miR-21, miR-29a, miR-126, and miR-218 did not differ according to the systemic extent of atherosclerosis to multiple territories (91).

## Summary

According to the four criteria in the “Mirnas As potential biomarkers of stable atherosclerosis” section, we selected a set of the most clinically relevant miRNAs in atherosclerosis of different territories and in multi-territorial atherosclerosis (Tables 1, 2).

**Table 1:** Most relevant miRNA associated with atherosclerosis of different territories.

	**Upregulated**	**Downregulated**
Coronary	miR-208, miR-215, miR-487a, miR-502	let-7, miR-29b, miR-145
Carotid	let-7, miR-21, miR-27a, miR-29a, miR-29c, miR-146a, miR-211, miR-218, miR-342-5p	miR-30, miR-31, miR-126-3p, miR-181b, miR-221, miR-320b.
Inferior limbs	miR-124, miR-1827, miR-4284, miR-4739	let-7, miR-15b, miR-16, miR-20b, miR-25, miR-26b, miR-27b, miR-28-5p, miR-126, miR-195, miR-221, miR-335, miR-363, miR-432, miR-4306, miR-4463
Renal	-	-
Different arterial territories	miR-21^λ^	miR-30^λ^, miR-126^α^, miR-145^β^, miR-221-3p^λ^,
Multi-territorial	-	miR-27b, miR-146a

α, Coronary, Carotid, Lower Limbs; β, Coronary, Carotid; λ, Carotid, Lower Limbs.

**Table 2:** Robustness of miRNA dysregulation in atherosclerosis of different territories.

**miRNAs**	**Reproducibility**	**Validation and derivation cohorts**	**Adjustment in multivariate analysis**	**Diagnostic accuracy**
**Coronary**				
miR-208		✓		✓
miR-215		✓		✓
miR-487a		✓		✓
miR-502		✓		✓
Let-7	✓		✓	✓
miR-29b		✓		✓
miR-145	✓			
**Carotid**				
Let-7, miR-30			✓	
miR-29c			✓	✓
miR-21	✓	✓	✓	✓
miR-27a, miR146a, miR-342-5p, miR-126-3p, miR-181b, miR-320b				✓
miR-31, miR-211, miR-218		✓		
miR-221	✓		✓	✓
**Lower limbs**				
miR-15b, miR-16, miR-363		✓		✓
miR-25, miR-28-5p, miR-126, miR-26b, miR-195, miR-335		✓		✓
miR-27b	✓	✓		✓
miR-124, miR-221	✓	✓		
miR-4739			✓	
let7e, miR-20b, miR-4284, miR-432, miR-4463, miR-4306, miR-1827		✓		

For each territory of atherosclerosis, the quality criteria that were met for each miRNA are presented with the symbol ✓.

In coronary atherosclerosis, the most relevant miRNAs include let-7, miR-29b and miR-208. Let-7 seems to be appropriate for the diagnosis considering that it was downregulated in different studies, the expression levels were adjusted in multivariate analysis and this miRNA showed reasonable diagnostic accuracy. Of note, a multiparameter approach, combining miR-487a, miR-502, miR-208, miR-215, and miR-29b presented a better diagnostic accuracy. On the other hand, miR-29b and miR-208 are also good candidates for diagnostic purposes since these miRNAs were validated in more than one cohort and, individually, presented the highest AUC.

In carotid atherosclerosis, the most relevant miRNAs include miR-21, miR-29c and miR-221. Both miR-21 and miR-221 had good reproducibility between different studies, were adjusted in the multivariate analysis, and had positive data regarding diagnostic accuracy. MiR-29c had a high AUC and its levels were adjusted in multivariate analysis.

In lower limb atherosclerosis the most relevant miRNAs are miR-27b, miR-15b, miR-16 and miR-363. While miR-27b had reproducibility in different studies, was validated in more than one cohort and presented reasonable diagnostic accuracy, miR-15b, miR-16, miR-363 were also validated in different cohorts and presented a higher diagnostic accuracy.

Regarding renal atherosclerosis, no major conclusions can be drawn considering the paucity of data.

The observed dysregulation of miR-27b and miR-146a according to the systemic severity of atherosclerosis and the accuracy of both miRNAs to predict multi-territorial atherosclerosis suggest that these miRNAs are potential diagnostic biomarkers for such a clinical scenario.

## Discussion

Understanding the molecular phenotypes of atherosclerosis may provide insights into its pathophysiology and contribute to identify biomarkers for use in clinical practice. Precise risk stratification tools, which can include circulating biomarkers, are important for cardiovascular risk prediction and the tailoring of treatment strategies, particularly for individuals in higher risk groups. In this review, we assessed the expression of circulating miRNAs in atherosclerosis of major arterial territories, in single- and multi-territorial disease. To the best of our knowledge, we provide the first critical review of circulating miRNA expression profiles in such clinical contexts.

Based on different criteria, we identified the most relevant miRNAs for atherosclerosis diagnosis. These results provide new insights into the role of miRNAs expression in atherosclerosis presentation in clinical practice and may consist of a non-invasive method. The potential diagnostic value of circulating miRNAs has been shown, which allows stratification of systemic atherosclerotic burden. Of note, screening of multi-territorial atherosclerosis in clinical practice by assessing simultaneously different arterial territories using currently available methods is potentially laborious. Therefore, a specific circulation miRNA signature could facilitate the early diagnosis of multi-territorial atherosclerosis.

Since premature atherosclerosis precedes the development of cardiovascular disease, identification of the associated biomarkers is of great importance. The identified miRNA profiles show a promising applicability as biomarkers in primary and secondary prevention of cardiovascular disease. They could be potentially useful for an early intensification of the atheroprotective regimens, such as antithrombotic and lipid-lowering therapies in patients with a higher atherosclerotic burden. Of note, the development of novel non-invasive biomarkers can improve the predictive capacity of current algorithms, beyond the traditional risk factors, as it was shown for some miRNAs.

The different circulating miRNA expression profiles described may also provide clues to pathophysiology of atherosclerosis. Although the pathophysiology of stable atherosclerosis of different territories is out of the scope of this review, the differential miRNA profiles suggest that there is a different pathophysiology of atherosclerosis in different territories. On the other hand, the common miRNA dysregulation in atherosclerosis of different territories suggests that there is a common pathophysiology in atherosclerosis irrespective of its location.

It was not appropriate to pool miRNA expression data since the methodologies of miRNAs analysis and presentation of results of miRNAs expression were heterogeneous and impaired the conversion to a common effect size. We present an objective assessment of their usefulness as diagnostic biomarkers based on four predefined criteria, including the diagnostic accuracy (area under the ROC curve), which are potentially meaningful for the clinical setting.

The selected key miRNAs are known to have a role in the atherosclerotic cascade. Theirs main functional relevance is here summarized.

### Let-7

Let-7 expression is critical for maintaining the integrity of endothelial cells (ECs). Let-7s are abundantly expressed in both ECs and VSMCs. In normal functioning, let-7 is responsible for transducing fibroblast growth factor (FGF) signaling into changes to TGF- β within endothelial cells, thereby limiting proliferation (92, 93). Let-7g has been shown to reduce endothelial cells inflammation and monocyte adhesion (94–97), diminish EC senescence, and play a role in controlling arterial stiffness and aging (98, 99). Moreover, has shown to be reduced in oxLDL treated VSMCs, and this reduction may partially be responsible to the oxLDL-induced cell proliferation and migration.

### miR-208

miR-208 acts on proteins associated with inflammation, endothelial apoptosis, VSMC proliferation and migration, including the *PPAR, ACTA2, ROR2* and *PI3K/AKT* pathways (100). miR-208b overexpression inhibits the expression of both *COL1* and *ACTA2* (the gene encoding smooth muscle α-actin) by directly targeting and inhibiting *GATA4* (101). Overexpression of *miR-208b* repress (102) and has a protective effect on blood vessels *via* ACTA2 inhibition (103).

### miR-21

miR-21 inhibits both endothelial cell apoptosis, through PTEN (104), and endothelial cell proliferation, through RhoB (105). It also promotes inflammatory activation of endothelial cells by targeting PPARα, which induces the expression of adhesion molecules and cytokines (106). Furthermore, enhances vascular smooth muscle cell migration and proliferation by targeting TSP-1 and c-Sk (107, 108), and reduces vascular inflammation by suppressing macrophage activity (109).

### miR-29b and miR-29c

miR-29 family is highly expressed in fibroblast and vascular smooth cells (VSMC). Besides playing a role in vascular homeostasis, it also regulates genes encoding collagen (*COL1A1, COL1A2, COL3A1*) and extracellular matrix proteins, including fibrillin (*FBN1*) and elastin (*ELN1*) (110).

### miR-221

miR-221 promotes VSMC proliferation by repressing the cyclin-dependent kinase inhibitor p27Kip1 and reduces expression of contractile genes (111). The miR-221 also controls differentiation of ECs but inhibits their proangiogenic activation, proliferation, and migration, so it is implicated in maintaining endothelial integrity and supporting quiescent EC phenotype (102). miR-221 stimulates neointima formation leading to atherogenic calcification of vascular smooth muscle cells (112).

### miR-27a and miR-27b

miR-27 was found to be concentrated in the cells associated with atherosclerotic lesions, such as human umbilical vein endothelial cells, smooth muscle cells and macrophage cells (113). In addition, miR-27 plays a key role in the cholesterol biosynthesis pathway, controlling the cholesterol efflux by reducing the expression of ABCA1 and apoA-1, and influx through targeting CD36 expression (114). In mice models, miR-27 regulated the expression of important metabolic genes, including angiopoietin-like 3 (ANGPTL3) and glycerol-3-phosphate acyltransferase 1 (GPAM) (114). miR-27b downregulates lipoprotein lipase gene expression and thereby reduces vascular inflammatory response (115). Moreover, miR-27b restrains the activity of NF-κB and the production of several proinflammatory factors (116). This contributes to a decreased monocyte–macrophage activation (39), which is atheroprotective as it results in decreased vascular inflammation (117). In addition, miR-27b also regulates angiogenesis through the angiogenic inhibitor semaphorin 6A and Notch ligand Dll4 (39, 118).

### miR-15b

miR-15b regulates cell proliferation, migration, and apoptosis by regulating the PI3K/AKT signaling pathway. It also significantly inhibited the expression of IGF1R in hVSMCs (119).

### miR-16

miR-16 has potent angiostatic effects by preventing VEGF signaling, EPC senescence, and endothelial proliferation. miR-16 targets VEGFR2 and FGR1 transcripts in endothelial cells (120) and directly silences de VEGF transcript (121, 122). Also, it was found that miR16 was highly expressed in contractile VSMCs by targeting the oncogene yes-associated protein (YAP) (123).

### miR-363

miR-363 is purported to be involved in the angiogenesis homeostasis, including regulation of endothelial cells, expression of angiogenesis factors and interactions between hematopoetic and endothelial cells (124). One study suggested that miR-363-3p, by targeting NOX4 *via* inactivation of the p38 MAPK signaling pathway, protects against endothelial cell injury caused by inflammation (125).

### miR-146a

miR-146a is induced in endothelial cells in response to proinflammatory cytokines and acts as a negative feedback regulator of inflammatory signaling in endothelial cells (126). It inhibits endothelial activation by promoting eNOS expression. On the other hand, the enhancement of miR-146a levels in monocytes and macrophages by cellular apoE was shown to suppress the NF-κB pathway and thus reduce the macrophage activity (127). Moreover, miR-146a targets the Toll-like receptor 4, reducing the formation of foam cells (46).

In addition to diagnosis role, in terms of therapeutics in the atherosclerosis field, the most successful RNA drugs in atherosclerosis are proprotein convertase subtilsin/kexin type 9 (PCSK9) inhibitors, a siRNA drug that targets PCSK9 mRNA (128). Inclisiran was approved in Europe in 2020 for use in adults with primary hypercholesterolaemia or mixed dyslipidaemia (129). Several pharmaceutical and biotech companies are working in the development of miRNA-based therapeutics, mainly miRNA mimics and antogomiRs. There are clinical trials focused on cancer, for example, targeting miR-155 in T-cell lymphoma (130); liver disease, as targeting miR-122 (Miravisen) in HCV infected hepatocytes (131, 132); or structural gastrointestinal diseases, for instance miRNA-124 as a target in inflammatory bowel disease (133). As the application of this biomarkers or intervention targets is increasing and miRNAs have proven to play an important role in vascular dysfunction, atherosclerotic disease should be a contemplated in future clinical trials given the high associated morbidity and mortality. In fact, there are currently some studies in the cardiovascular field. Still in preclinical stage are MGN-1374 (targets: miRNA-15 and miR-195) for the treatment of post-myocardial infarction, MGN-2677 (targets: miR-143/145) for the treatment of vascular disease, MGN-4220 (target: miR-29) for the treatment of cardiac fibrose, MGN-5804 (target: miR-378) for the treatment of cardiometabolic disease, MGN-6114 (target: miR-92) for the treatment of peripheral arterial disease and MGN-9103 (target: miR-208) for the treatment of chronic heart failure. MRG-110 (target: miR-92a) to target blood vessel growth and to control ischemia is in phase-I clinical trial (134, 135). Besides this last one and PCSK9 inhibitors, also IONIS-ANGPTL3-L_RX_ (target: hepatic ANGPTL3 mRNA; NCT02709850) and AKCEA-APOCIII-LRX (target: hepatic APOC3 mRNA; NCT02709850) are in development in the atherosclerosis field. Furthermore, the emerging field on nanomedicine shows promising possibilities for using miRNA which can enhance treatment options.

Given this, panels of the miRNAs identified previously for each atherosclerosis location could be used for diagnosis and the integration of the expression levels could be used for preventive therapy and follow-up.

### Limitations

The identification of dysregulated miRNAs according to different arterial territories, based on available data, has some limitations. Most studies did not effectively exclude atherosclerosis in other territories other than the index territory. Moreover, a direct comparison of atherosclerosis of different territories has not been carried out in the same study and inferences were made using studies focused on different territories. In fact, the samples may have differed across studies and the differences in miRNAs expression may be attributable to unmeasured confounders, including clinical, demographic, and methodological parameters. Of note, there was a heterogeneity of technical approaches for miRNA assessment. Further prospective studies are needed to directly compare the miRNA profiles in atherosclerosis of different arterial territories.

## Conclusion

miRNAs regulate different pathways associated with stable atherosclerosis initiation, progression, and expression, and circulating miRNAs are potential biomarkers of stable atherosclerosis. Atherosclerosis of different territories is associated with specific circulating miRNA profiles, including the coronary, carotid, and lower limbs single-territorial atherosclerosis. There is also a common circulating miRNA profile in atherosclerosis irrespective of its location and a miRNA signature in multi-territorial atherosclerosis. These miRNAs may consist of non-invasive biomarkers of atherosclerosis of different territories in clinical practice.

## Author contributions

AT: conceptualization and writing. VF: conceptualization. TP-d-S: conceptualization and reviewing. RF: supervision. All authors contributed to the article and approved the submitted version.

## Conflict of interest

The authors declare that the research was conducted in the absence of any commercial or financial relationships that could be construed as a potential conflict of interest.

## Publisher's note

All claims expressed in this article are solely those of the authors and do not necessarily represent those of their affiliated organizations, or those of the publisher, the editors and the reviewers. Any product that may be evaluated in this article, or claim that may be made by its manufacturer, is not guaranteed or endorsed by the publisher.
